# Demand and Signing of General Practitioner Contract Service among the Urban Elderly: A Population-Based Analysis in Zhejiang Province, China

**DOI:** 10.3390/ijerph14040356

**Published:** 2017-03-29

**Authors:** Yanrong Zhao, Junfen Lin, Yinwei Qiu, Qing Yang, Xinyi Wang, Xiaopeng Shang, Xiaoping Xu

**Affiliations:** Zhejiang Provincial Center for Disease Control and Prevention, 3399 Binsheng Road, Binjiang District, Hangzhou 310051, China; yrzhao@cdc.zj.cn (Y.Z.); jflin@cdc.zj.cn (J.L.); ywqiu@cdc.zj.cn (Y.Q.); qyang@cdc.zj.cn (Q.Y.); xywang@cdc.zj.cn (X.W.); xpshang@cdc.zj.cn (X.S.)

**Keywords:** health service needs and demands, general practitioners, the elderly

## Abstract

This study aims to examine whether the urban elderly in the Zhejiang Province of China signed contracts with their general practitioner (GP) based on their health service needs, and to further identify the determinants of their demand and signing decisions. A community-based cross-sectional study was conducted in 16 community health service (CHS) institutions in Zhejiang Province, China. The urban elderly over 60 years of age were enrolled when visiting the sampled CHS. Baseline characteristics were compared between participants using Chi-Square tests for categorical variables. Univariate and multivariable logistic regression analyses were used to identify determinants of the GP contract service demand and signing decisions, respectively. Among the 1440 urban elderly, 56.67% had signed contracts with their GP, and 55.35% had a demand of the GP contract service. The influencing factors of demand were a history of diabetes or cardiovascular disease (OR = 1.33, 95% CI, 1.05–1.68); urban resident basic medical insurance (URBMI) vs. urban employee basic medical insurance (UEBMI) (OR = 1.96, 95% CI, 1.46–2.61); and middle-income vs. low-income (OR = 0.67, 95% CI, 0.50–0.90 for RMB 1001–3000; OR = 0.59, 95% CI, 0.39–0.90 for RMB 3001–5000). Having a demand for the GP contract service was the strongest determinant of signing decisions (OR = 13.20, 95% CI, 10.09–17.27). Other factors also contributed to these decisions, including gender, caregiver, and income. The urban elderly who had signed contracts with GPs were mainly based on their health care needs. Elderly people with a history of diabetes or cardiovascular disease, as well as those with URBMI, were found to have stronger needs of a GP contract service. It is believed that the high-income elderly should be given equal priority to those of low-income.

## 1. Introduction

With the effective control of infectious diseases and an aging population, China has experienced an epidemiological transition shifting from infectious to chronic diseases in a much shorter time than many other countries. The nationwide pooled prevalence of hypertension, heart disease, cerebral vascular disease, and diabetes was 96.2‰ in 2008, increasing steadily from 33.3‰ in 1993 [1]. A general medicine model which is prevention and community oriented has become more and more important. Simultaneously, the Chinese government is faced with widespread public discontent stemming from the government’s neglect of the primary health-care system [2]. The central Chinese government reduced its budget on health care expenses, especially during the early years of the economic reforms launched in the early 1980s. In response, local governments changed their focus from primary care to hospital-based specialty care. In 2007, more than 85.0% of China’s health care resources were allocated to these higher-level care facilities, whereas less than 15.0% were reserved for primary care facilities [3].

Under this background, China unveiled its health-care reform plan in April 2009 with the goal of providing affordable and equitable basic health care for all by 2020 [2]. In the planned model, primary-care centers will take on a gate-keeping role through the hierarchical medical system and bidirectional referral mechanisms between primary-care centers and secondary or tertiary hospitals. Patients receive health care services at primary-care centers for the most common and minor illnesses, and are only referred to hospitals for more complex problems [4]. The central government proposed the establishment of a general practitioner (GP) system to carry out contract service in 2011, which has strengthened the importance of primary-care centers in the national health strategy, and is regarded as the foundation of the hierarchical medical system and bidirectional referral mechanism. In the mode of contract service, residents can choose their GPs voluntarily from local primary-care centers, such as community health service institutions (CHS) in urban areas. A resident can only sign a health service contract with one GP, while one GP can sign as many contracts with residents as is appropriate based on their own capacity, or that of the GP team. The contracts remain in force for at least one year. If the resident is not satisfied with the GP, he/she has the right to choose another one for the next year. The GP is contracted to provide basic medical and public health services free of charge (such as free basic diagnosis and treatment fees) to signed residents, as well as personalized health management services (such as home visits or home care beds) at a discounted price, if required [5]. Meanwhile, the residents are encouraged to visit their signed GPs when first feeling discomfort (except for emergencies), and are referred to a specialist at secondary or tertiary hospitals only when the GP believes it is necessary [6]. After treatment in secondary or tertiary hospitals, residents return to their GP to receive follow-up treatment or rehabilitation.

General medicine has developed since the introduction of general medicine to China in the 1980s, and the cultivation of GPs has also made progress. The Chinese central government sped up the development of GPs since the health care reform in 2009, and announced a goal of providing two to three GPs for every 10,000 people by 2020 (around 300,000 GPs) in 2011 [7]. In 2012, the national ratio was only 0.81 (the eastern, central, and western regions were 1.19, 0.52, and 0.58, respectively)—significantly lower than 2.24 in Zhejiang Province [1]. Furthermore, while the GP contract service was only carried out in 10 pilot cities at the national level [8], it was already carried out widely at the county level in Zhejiang in 2012 [9]. The signing rate of the surveyed residents in 34 counties of China was 38.40% in 2014, with higher rates in residents with hypertension or diabetes (42.91% and 45.38%, respectively) [8]. The factors influencing residents’ contract behavior were various in different reports; however, advanced age was commonly proven to be related with higher signing rate [10,11]. In Zhejiang Province, the contract prioritizes vulnerable people, such as the elderly aged over 60 years. According to data reported in Zhejiang Province, there were 4,288,833 elderly people who had signed contracts with their GPs, with a signing rate of 47.02% in 2015—significantly higher than the 19.92% reached in 2013 [12].

Despite these encouraging results, the question of whether residents who had signed contracts did so based on their health care needs required further study. Previous studies in China found that there were residents who had signed contracts only because of the free policy [13]. We wanted to know if they had signed contracts without utilizing the health services, and what had influenced their use of these services. Data from previous studies showed that advanced age (especially frail elderly people), income, cohabitation status (absence of a spouse or living alone), and bad physical health status were related to receiving in-home (long term) care or care requirements [14,15]. Gender difference remained controversial in previous studies [14], and little was known about the determinants behind signing the GP contract service and the demand for these services in China, especially among the urban elderly in Zhejiang Province. The objectives of this study were to examine whether the urban elderly signed contracts with their GP based on their real health service needs, and to further identify the determinants of their demand and signing decision.

## 2. Materials and Methods

### 2.1. Study Design and Participants

A community-based cross-sectional study was conducted through a face-to-face survey in Zhejiang Province, China in September 2015. A multilevel stratified random sampling method was employed to select 8 out of 90 counties based on geographical position, economy development, and the evaluation results of basic public health service ability [16], and two CHS institutions from each sampled county (average 16 CHS institutions per county) were selected randomly. Elderly people over 60 years were eligible for enrolment when visiting the selected CHS institutions for medical or health management service. The young-elderly (60–74 years) and old-elderly (≥75 years) were both investigated out of a sample size of about 50 in each CHS institution. The impermanent residents of the sampled community were excluded. If there were two or more eligible elderly people from one family, only one was randomly selected to participate in the survey.

### 2.2. Measures

The primary outcomes in this study were demand and the signing decision of the GP contract service. The GP contract service demand was categorized as yes, no, and uncertain. The GP contract service signing stood for a formally signed contract between an individual and a GP in a CHS institution.

Covariates included demographic, socioeconomic, and health characteristics. Age was reported in years, while other covariates were reported in categories such as sex, caregiver, medical insurance type, personal income, health status, and chronic disease history. The caregivers category consisted of spouse, children/grandchildren, others (such as community worker), and none. Medical insurance types were categorized as urban employee basic medical insurance (UEBMI), urban resident basic medical insurance (URBMI), and others (such as private insurance). Income was investigated based on the average personal income per month (RMB 1000 and less; RMB 1001–3000; RMB 3001–5000; and RMB 5001 and above). Self-evaluated health status was measured using a four-level scale from very good to very bad. Ten chronic diseases were examined in the study, including hypertension, diabetes, coronary heart disease, cerebral vascular disease, intervertebral diseases, rheumatoid arthritis, gastroenteritis, cholecystitis and gallstones, cataracts, and chronic obstructive pulmonary disease (COPD).

### 2.3. Data Collection

A standard questionnaire was used to collect information. The face-to-face interview was conducted by well-trained local CHS doctors and centers for disease control and prevention (CDC) staff. The information received from the question “Did you sign with a GP?” was checked against the contract records held in CHS institutions.

### 2.4. Statistical Analysis

Baseline characteristics were compared between participants using Chi-Square tests for categorical variables. To identify the factors influencing the GP contract service demand, univariate and multivariable logistic regression analyses were used to estimate the odds ratio (OR) and 95% confidence intervals (CIs), respectively, including sex, age group, caregiver, self-evaluated health status, chronic disease history, medical insurance type, and personal income. To examine the determinants behind the signing decision, we added the GP contract service demand as well as other covariates in the model. All analyses were performed with the SPSS (Statistical Package for the Social Sciences), version 13.0 (IBM, Chicago, IL, USA). A *p* < 0.05 was accepted as indicating statistical significance.

### 2.5. Ethical Statement

Research protocols were approved by Zhejiang Provincial Center for Disease Control and Prevention (Ethic approval code: T-043-R). All subjects provided written informed consent after the research protocols were carefully explained to them, and the informed consent of all participants were received. 

## 3. Results

### 3.1. Sample Characteristics

Of the 1447 elderly people who accepted our invitation to participate in the study, seven of them were unable to provide key information (lack of information on “the GP contract service demand” or “Did you sign with the GP?”), which meant that only data from 1440 participants were analyzed. The mean age was 72.74 years (SD 7.64 years), with the majority of them being female (60.76%).

There were 56.67% elderly people who had signed contracts with their GPs and 55.35% who thought that they needed the GP contract service. Compared with the elderly who had not signed contracts with their GP, those who had signed were more likely: to be older, have either a history of diabetes or cardiovascular disease, have URBMI medical insurance, have less spousal care, or have a relatively low-income. A similar trend was seen in the comparison of the elderly who thought they needed the GP contract service and those who did not (Table 1).

It is worth noting that 20.34% of elderly people who signed with a GP did not think that they would need the service, while 23.56% of elderly people who did not sign with a GP thought that they needed the service.

### 3.2. Factors Influencing the GP Contract Service Demand of the Elderly

According to the univariate logistic regression analysis results, the elderly with the following factors seemed to have a stronger demand of the GP contract service, including age over 80 years: non-family member care, a history of diabetes or cardiovascular disease, and having URBMI medical insurance. The elderly in the middle-income bracket seemed to be less in need of the GP contract service. Sex, different chronic disease history, and self-evaluated health status were not influencing factors.

The multivariable logistic regression analysis showed that a history of diabetes or cardiovascular disease was associated with a stronger demand (OR = 1.33, 95% CI, 1.05–1.68). Medical insurance type was another factor that influenced demand, with those insured by the URBMI having a higher demand (OR = 1.96, 95% CI, 1.46–2.61). Compared with the low-income elderly, those in the middle-income level had lower demand (OR = 0.67, 95% CI, 0.50–0.90 for RMB 1001–3000; OR = 0.59, 95% CI, 0.39–0.90 for RMB 3001–5000), while those in a high-income bracket had similar demands for the service (OR = 1.19, 95% CI, 0.67–2.13). The demands of the elderly in various age groups were not significantly different, nor were those who had different caregivers (Table 2).

### 3.3. Factors Influencing the GP Contract Service Signing Decision of the Elderly

The univariate and multivariable logistic regression analyses both showed that a demand for the GP contract service was the strongest determinant of the GP contract service signing decision (OR = 13.20, 95% CI, 10.09–17.27).

Therefore, the factors which influenced their demand would also influence their signing decision, as shown in the univariate analysis results such as age group, caregiver, a history of diabetes or cardiovascular disease, medical insurance type, and income. According to the multivariable analysis results (which controlled the confounding influence of demand), there were further factors which contributed to their signing decisions, such as gender, caregiver, and income. The elderly taken care of by their children/grandchildren or themselves were more willing to sign a contract with their GP; however, those who were female and in a high-income level were less willing to do so (Table 3).

## 4. Discussion

Experience from developed countries (especially the U.S. and the UK) where primary care is provided mainly by family doctors or general practitioners with standardized postgraduate training is encouraging [17,18,19,20]. For people with regular family doctors, primary care is most effective in gate-keeping secondary care [21]. The family-doctor model has been proven to effectively reduce the nursing institutions and hospital admission rate, as well as improve physical function and maintain independent living in elderly people [22,23,24].

China initiated its CHS program to promote primary health care in 1997 [25]; however, the central government did not propose the establishment of the GP system until 2011 [26], which is still in its initial stage. General practice is new in China and faces a range of challenges. Patients used to seek care at large hospitals for simple health problems because of their low trust in the CHS, not only due to the poor care received from GPs, but also the outdated equipment [2,6,25]. China has done much to create a favorable environment for the growth of the GP system, including several training programs to improve the competency of the less-educated doctors and nurses in the CHS to cultivate GPs [7,27]. As mentioned previously, the government and health administrative department of Zhejiang Province has also actively promoted the GP contract service in recent years, which has seen the contract signing rate in Zhejiang Province increase yearly. As mentioned earlier, the elderly have more opportunities to sign contracts with their GP [10,11]; therefore, under the background of limited medical resources, the elderly (especially those with chronic diseases) are always given priority to the GP contract service in China, including Zhejiang province.

Based on our study, the signing rate of the GP contract service in the urban elderly was 56.67%, close to the demand rate (55.35%), and a bit higher than the report signing rate in the entire elder population of Zhejiang province (47.02%). The strongest association was found between the “signing decision with the GP” and “demand of the GP contract service”. It indicated that the urban elderly in Zhejiang Province signed contracts with their GPs based mainly on their real health care needs. A history of diabetes or cardiovascular disease, medical insurance type, and income were all determinants of the demand. However, our study also found that 20.34% of the elderly who signed contracts with the GP thought that they might not need the service, which was in contrast to the 23.56% of elderly who did not sign contracts with their GP, but thought that they really needed the service. In addition to the demand, there were other factors that affected the signing decision, such as gender, caregiver, and income. It is worth considering that income had different effects on demand and the signing decision. Compared to those of middle-income, elderly people with low- (≤RMB 1000 per month) and high- (≥RMB 5001 per month) income had higher demands of the service; however, those with higher income (RMB 3001–5000 or ≥RMB 5001 per month) were less likely to sign contracts (the median annual disposable income of Chinese urban residents was RMB 29,129 in 2015 [28], equivalent to RMB 2427 per month). This could be due to vulnerable groups being given first priority in signing with the GP contract service, such as those with low-income, poor medical insurance (URBMI), bad physical health, or living alone. The others may have been easily ignored in the signing program, such as those with high-income, good medical insurance (UEBMI), good health, and living with a spouse. Compared with the UEBMI, the URBMI has a low premium and low reimbursement rate (UEBMI: total premium per person was RMB 1559 and the inpatient reimbursement rate was 68.2% with an individual contribution of RMB 494–741; URBMI: total premium per person was RMB 138 with an inpatient reimbursement rate of 47.9% and an individual contribution of RMB 20–250) [2]. In reality, the high-income elderly had better health awareness, tended to agree with advanced health service ideas, and had a higher demand of the GP contract service. Therefore, they deserve the same access to the contract service as those with low-income.

Compared with secondary or tertiary hospitals, the CHS institutions have unique advantages in the health management of the elderly—especially those with diabetes or cardiovascular disease. These diseases usually have a long course and need long-term medication or rehabilitation. When a diagnosis is confirmed, patients prefer to go to the CHS to take medication and receive simple laboratory tests (measurement of plasma glucose or blood pressure, and so on), where it is much more convenient than the secondary or tertiary hospitals. Patients signed with a GP can also receive personalized services such as home visits or home care beds, which is impossible in the secondary or tertiary hospitals. Since 2009, the Chinese government has provided RMB 15 per head (raised to RMB 45 in 2016, with future increases promised) for primary health-care providers to deliver a defined package of basic public health services, which is included as part of the GP services [2]. Hypertension and diabetes management are key parts of the service package; for example, diabetes management includes a yearly clinical assessment, a quarterly plasma glucose assessment, education on healthy diets, physical activity, and medication adherence, and routine follow-up visits [29]. This could be why the elderly with a history of diabetes or cardiovascular disease have significantly higher demands on the GP contract service.

Age was previously found to be a strong and consistent predictor of health care needs [14,15]; however, we did not find the same evidence in our study, which confined the study population to only the urban elderly. Although the gender difference in demand was not found to be significant in our study, females were less likely to sign with a GP. More studies are needed to explain this phenomenon.

## 5. Limitations

The participants were recruited when they visited the randomly sampled CHS institutions in an urban area for medical or health management service; therefore, the study sample was not fully representative of the urban elderly. For instance, more low-income elderly could be captured as higher-income people might prefer tertiary hospitals, which limits the extrapolation of the findings. Furthermore, the signing and demand rate of the GP contract service could be overestimated, as the investigation was carried out in CHS institutions.

## 6. Conclusions

Despite the significantly increasing signing rate of GP contract services, the GP system is still in its initial stage in Zhejiang province, China. In addition to the political impetus, the urban elderly who signed contracts with their GP did so based mainly on their health care needs. The urban elderly with a history of diabetes or cardiovascular disease, and those with the URBMI were found to have a stronger need of the GP contract service; therefore, they should be given first priority to the service. Contrary to popular belief, the high-income elderly also deserve equal priority to those with low-income, and age may not be an factor influencing health care needs when the study population is confined to only the urban elderly.

## Figures and Tables

**Table 1:** General characteristics of study participants.

Characteristics	Signed with a GP				GP Contract Service Demand			
	Yes (*n* = 816)	No (*n* = 624)	Total (*n* = 1440)	*p* Value	Yes (*n* = 797)	No/Uncertain (*n* = 643)	Total (*n* = 1440)	*p* Value
Sex, %				0.09				0.72
Male	41.18	36.70	39.24		39.65	38.72	39.24	
Female	58.82	63.30	60.76		60.35	61.28	60.76	
Age group, %				0.03				0.02
60–	36.03	41.03	38.19		35.88	41.06	38.19	
70–	40.20	39.26	39.79		40.28	39.19	39.79	
80–	23.77	19.71	22.01		23.84	19.75	22.01	
Caregiver, %				<0.001				0.10
Spouse	54.78	66.35	59.79		58.09	61.90	59.79	
(Grand)children	28.43	23.40	26.25		27.35	24.88	26.25	
Others (e.g., community worker)	3.19	2.56	2.92		3.76	1.87	2.92	
None	13.60	7.69	11.04		10.79	11.35	11.04	
Chronic disease history, %				0.58				0.41
Yes	83.95	82.85	83.47		84.19	82.58	83.47	
No	16.05	17.15	16.53		15.81	17.42	16.53	
Diabetes or cardiovascular disease history, %				0.03				0.04
Yes	71.08	65.54	68.68		70.89	65.94	68.68	
No	28.92	34.46	31.32		29.11	34.06	31.32	
Self-evaluated health status, %				0.58				0.35
Very good	28.94	27.17	28.17		29.04	27.10	28.17	
Good	49.69	52.09	50.74		50.19	51.40	50.74	
Bad	20.62	18.97	19.90		20.00	19.78	19.90	
Very bad	0.75	1.77	1.19		0.76	1.71	1.19	
Medical insurance, %				<0.001				<0.001
UEBMI	35.17	57.69	44.93		35.88	56.14	44.93	
URBMI	63.60	41.51	54.03		62.99	42.92	54.03	
Others	1.23	0.80	1.04		1.13	0.93	1.04	
Average personal income per month, %				<0.001				<0.001
≤RMB 1000	29.90	17.79	24.65		30.61	17.26	24.65	
RMB 1001–3000	50.49	44.07	47.71		46.80	48.83	47.71	
RMB 3001–5000	14.83	31.89	22.22		16.69	29.08	22.22	
≥RMB 5001	4.78	6.25	5.42		5.90	4.82	5.42	

GP: general practitioner; UEBMI: urban employee basic medical insurance; URBMI: urban resident basic medical insurance.

**Table 2:** Factors influencing the GP contract service demand by univariate and multivariable logistic regression analysis.

Influence Factor	Univariate		Multivariable ^c^	
	Odds Ratio (95% CI)	*p* Value	Odds Ratio (95% CI)	*p* Value
Sex				
Male	1.00 (reference)		-	-
Female	0.96 (0.78–1.19)	0.72	-	-
Age group				
60–	1.00 (reference)		-	-
70–	1.18 (0.93–1.49)	0.18	-	-
80–	1.38 (1.04–1.83)	0.02	-	-
Caregiver				
Spouse	1.00 (reference)		-	-
(Grand)children	1.17 (0.92–1.50)	0.20	-	-
Others (e.g., community worker)	2.15 (1.09–4.25)	0.03	-	-
Nobody	1.01 (0.72–1.42)	0.94	-	-
Chronic diseases history ^a^				
No	1.00 (reference)		-	-
Yes	1.12 (0.85–1.48)	0.41	-	-
Diabetes or cardiovascular disease history ^b^				
No	1.00 (reference)		1.00 (reference)	
Yes	1.26 (1.01–1.57)	0.04	1.33 (1.05–1.68)	0.02
Self-evaluated health status				
Very bad	1.00 (reference)		-	-
Bad	2.27 (0.82–6.30)	0.12	-	-
Good	2.19 (0.80–5.98)	0.13	-	-
Very good	2.40 (0.87–6.62)	0.09	-	-
Medical insurance ^c^			-	-
UEBMI	1.00 (reference)		1.00 (reference)	
URBMI	2.30 (1.85–2.84)	<0.001	1.96 (1.46–2.61)	<0.001
Average personal income per month (USD$:RMB¥ = 1:6.9)				
≤RMB 1000	1.00 (reference)		1.00 (reference)	
RMB 1001–3000	0.54 (0.41–0.71)	<0.001	0.67 (0.50–0.90)	0.01
RMB 3001–5000	0.32 (0.24–0.44)	<0.001	0.59 (0.39–0.90)	0.01
≥RMB 5001	0.69 (0.42–1.14)	0.15	1.19 (0.67–2.13)	0.55

^a^ Participants with any history of the following diseases: hypertension, diabetes, coronary heart disease, cerebral vascular disease, intervertebral diseases, rheumatoid arthritis, gastroenteritis, cholecystitis and gallstones, cataracts, and chronic obstructive pulmonary disease. ^b^ Participants with any history of the following diseases: hypertension, diabetes mellitus, coronary heart disease, and cerebral vascular disease. ^c^ Excludes 15 participants of other medical insurance types.

**Table 3:** Factors influencing the GP contract service signing decision by univariate and multivariable logistic regression analysis.

Influence Factor	Univariate		Multivariable ^c^	
	Odds Ratio (95% CI)	*p* Value	Odds Ratio (95% CI)	*p* Value
Sex				
Male	1.00 (reference)		1.00 (reference)	-
Female	0.83 (0.67–1.03)	0.09	0.53 (0.40–0.71)	<0.001
Age group				
60–	1.00 (reference)		-	-
70–	1.17 (0.92–1.48)	0.20	-	-
80–	1.37 (1.04–1.82)	0.03	-	-
Caregiver				
Spouse	1.00 (reference)		1.00 (reference)	-
(Grand)children	1.47 (1.15–1.88)	<0.01	1.62 (1.19–2.22)	<0.01
Others (e.g., community worker)	1.51 (0.80–2.85)	0.21	0.84 (0.37–1.89)	0.67
Nobody	2.14 (1.49–3.08)	<0.001	3.08 (1.89–4.78)	<0.001
Chronic diseases history ^a^			-	
No	1.00 (reference)		-	-
Yes	1.08 (0.82–1.43)	0.58	-	-
Diabetes or cardiovascular disease history ^b^				
No	1.00 (reference)		-	
Yes	1.29 (1.03–1.62)	0.03	-	0.02
Self-evaluated health status			-	
Very bad	1.00 (reference)			-
Bad	2.58 (0.93–7.17)	0.07	-	-
Good	2.26 (0.83–6.19)	0.11	-	-
Very good	2.53 (0.92–6.97)	0.07	-	-
Medical insurance ^c^			-	-
UEBMI	1.00 (reference)		-	
URBMI	2.51 (2.03–3.12)	<0.001	-	<0.001
Average personal income per month (USD$:RMB¥ = 1:6.9)			-	
≤RMB 1000	1.00 (reference)		1.00 (reference)	
RMB 1001–3000	0.68 (0.52–0.89)	0.001	0.89 (0.64–1.24)	0.49
RMB 3001–5000	0.28 (0.20–0.38)	<0.001	0.34 (0.23–0.51)	<0.001
≥RMB 5001	0.46 (0.28–0.75)	<0.001	0.39 (0.21–0.73)	<0.01
GP contract service demand				
No	1.00 (reference)		1.00 (reference)	
Yes	12.71 (9.88–16.34)	<0.001	13.20 (10.09–17.27)	<0.001

^a^ Participants with any history of the following diseases: hypertension; diabetes; coronary heart disease; cerebral vascular disease; intervertebral diseases; rheumatoid arthritis; gastroenteritis; cholecystitis and gallstones; cataracts; and chronic obstructive pulmonary disease. ^b^ Participants with any history of the following diseases: hypertension, diabetes mellitus, coronary heart disease, and cerebral vascular disease. ^c^ Excludes 15 participants of other medical insurance types.
